# A Mobile Health Lifestyle Program for Prevention of Weight Gain in Young Adults (TXT2BFiT): Nine-Month Outcomes of a Randomized Controlled Trial

**DOI:** 10.2196/mhealth.5768

**Published:** 2016-06-22

**Authors:** Margaret Allman-Farinelli, Stephanie Ruth Partridge, Kevin McGeechan, Kate Balestracci, Lana Hebden, Annette Wong, Philayrath Phongsavan, Elizabeth Denney-Wilson, Mark F Harris, Adrian Bauman

**Affiliations:** ^1^ School of Life and Environmental Sciences Charles Perkins Centre University of Sydney University of Sydney Australia; ^2^ School of Public Health University of Sydney University of Sydney Australia; ^3^ School of Public Health Charles Perkins Centre University of Sydney University of Sydney Australia; ^4^ School of Nursing University of Technology Sydney Sydney Australia; ^5^ Centre for Primary Health Care and Equity University of New South Wales Kensington Australia

**Keywords:** young adult, weight gain prevention, mHealth, telehealth, fruit, vegetables, take-out foods, sugar-sweetened beverages, physical activity

## Abstract

**Background:**

The unprecedented rise in obesity among young adults, who have limited interaction with health services, has not been successfully abated.

**Objective:**

The objective of this study was to assess the maintenance outcomes of a 12-week mHealth intervention on prevention of weight gain in young adults and lifestyle behaviors at 9 months from baseline.

**Methods:**

A two-arm, parallel, randomized controlled trial (RCT) with subjects allocated to intervention or control 1:1 was conducted in a community setting in Greater Sydney, Australia. From November 2012 to July 2014, 18- to 35-year-old overweight individuals with a body mass index (BMI) of 25-31.99 kg/m2 and those with a BMI ≥ 23 kg/m2 and a self-reported weight gain of ≥ 2 kg in the past 12 months were recruited. A 12-week mHealth program “TXT2BFiT” was administered to the intervention arm. This included 5 coaching calls, 96 text messages, 12 emails, apps, and downloadable resources from the study website. Lifestyle behaviors addressed were intake of fruits, vegetables, sugar-sweetened beverages (SSBs), take-out meals, and physical activity. The control group received 1 phone call to introduce them to study procedures and 4 text messages over 12 weeks. After 12 weeks, the intervention arm received 2 further coaching calls, 6 text messages, and 6 emails with continued access to the study website during 6-month follow-up. Control arm received no further contact. The primary outcome was weight change (kg) with weight measured at baseline and at 12 weeks and self-report at baseline, 12 weeks, and 9 months. Secondary outcomes were change in physical activity (metabolic equivalent of task, MET-mins) and categories of intake for fruits, vegetables, SSBs, and take-out meals. These were assessed via Web-based surveys.

**Results:**

Two hundred and fifty young adults enrolled in the RCT. Intervention participants weighed less at 12 weeks compared with controls (model β=−3.7, 95% CI −6.1 to −1.3) and after 9 months (model β=− 4.3, 95% CI − 6.9 to − 1.8). No differences in physical activity were found but all diet behaviors showed that the intervention group, compared with controls at 9 months, had greater odds of meeting recommendations for fruits (OR 3.83, 95% CI 2.10-6.99); for vegetables (OR 2.42, 95% CI 1.32-4.44); for SSB (OR 3.11, 95% CI 1.47-6.59); and for take-out meals (OR 1.88, 95% CI 1.07-3.30).

**Conclusions:**

Delivery of an mHealth intervention for prevention of weight gain resulted in modest weight loss at 12 weeks with further loss at 9 months in 18- to 35-year-olds. Although there was no evidence of change in physical activity, improvements in dietary behaviors occurred, and were maintained at 9 months. Owing to its scalable potential for widespread adoption, replication trials should be conducted in diverse populations of overweight young adults.

**Trial Registration:**

Australian and New Zealand Clinical Trials Registry (ANZCTR): ACTRN12612000924853; (Archived by WebCite at http://www.webcitation.org/6i6iRag55)

## Introduction

The World Health Organization (WHO) declared a global obesity epidemic in 1998, but to date, progress in reversing or even halting increases in prevalence has failed [1]. There is some evidence that the rise in childhood obesity has plateaued in countries such as the United States and Australia [2]. However, young adulthood is an important population group that has been largely neglected and their steep trajectory of weight gain is mostly unrecognized [3,4]. This is of concern as incident obesity at a younger age carries increased risk of mortality from and morbidities of cardiovascular disease, type 2 diabetes, some cancers, and osteoarthritis among others [5-8]. Failure to address the weight gain of young adults may limit the success of childhood prevention programs.

Systematic literature reviews in recent years have drawn attention to the limited evidence base for successful interventions in 18- to 35-year-olds [9]. Many studies have had small numbers of subjects but have shown no potential for translation and scale-up to the community [10]. Rather, the use of young adults as subjects has been coincidental as college-based researchers find it easy to recruit on campus [11].

The life cycle phase termed “emerging adulthood” (18-24 years) signifies the transition from adolescence to leaving school, going to college or finding a job, and increasing independence [12]. It has been identified that this might be a window of opportunity to improve health behaviors as they become more receptive as the rebellion of teenage years is left behind [13]. Young adults need healthy lifestyles to both avoid obesity-related diseases in middle age and to protect their future progeny [14-16].

In 2010, the United States acknowledged young adults as a group requiring intervention for the prevention of weight gain with the award of research funding for the seven EARLY studies targeting 18- to 35-year-olds. [17]. Decreases in physical activity and continued high consumption of sugar-sweetened beverages (SSBs) and food prepared outside home are common lifestyle behaviors in young adults across many western nations [18]. With almost universal ownership of mobiles phones, (91% of young adults in the United States and 95% in Australia), this communication channel could be exploited for intervention delivery, referred to as mHealth [19]. Mobile phones have many features that can be used to provide education and counseling, such as text messaging, apps, and Internet access, in addition to the traditional voice call function.

Here we describe the effectiveness of a 9-month randomized controlled trial (RCT) of an mHealth program for 18- to 35-year-olds conducted in Australia, which aimed to improve lifestyle behaviors [20]. We hypothesized that those overweight young adults who received our 12-week “TXT2BFiT” program followed by a 6-month low-dose maintenance phase would gain less weight compared with those who received minimal intervention.

## Methods

### Study Population

Participants were aged 18-35 years and lived in Greater Sydney, Australia. All participants provided written informed consent and the study was approved by the institutional human ethics review board [20]. A detailed description of the recruitment process has been published previously [21]. In brief, subjects were recruited using mailings from primary care physicians, print media including posters, mass delivery of brochures and newspaper advertisements, and electronic media [21]. No racial or gender bias existed in the recruitment process.

### Study Design

The study was registered with the Australian and New Zealand Clinical Trials Registry (ACTRN12612000924853) and the study protocol published beforehand [20]. This study is a parallel two-arm RCT with subjects randomized 1:1. The deviation from the original protocol was that recruitment was extended from letters of invitation sent from primary care practices to include print and electronic media advertisements [21]. Regardless of recruitment method, all participants were required to visit a primary care physician to enter the study. Only 250 participants were recruited (due to slower-than-expected recruitment) rather than the 354 participants in the protocol [21].

Enrolment took place from November 2012 until July 2014. Participants completed an online screener to assess eligibility and those who met inclusion criteria attended a paid consultation with a primary care physician to verify medical fitness to participate. Participants were then allocated to one of two study arms, control or “TXT2BFiT” intervention, using a stratified block design according to sex and the primary care practice responsible for confirming eligibility. The randomized block contained block sizes 2, 4, and 6. A randomization list was generated using RAND.exe [22] and held centrally by the statistician. When an individual was recruited, the statistician assigned the treatment arm but was blind to intervention status. Other researchers involved in measurement and analysis were also blinded. Participants had their treatment arm concealed but were aware of the intensity of intervention received [20].

Eligibility criteria included age between 18 and 35 years, a body mass index (BMI) of 25 to 31.9 kg/m^2^, or 23 to 24.9 kg/m^2^ and > 2 kg self-reported weight gain in preceding 12 months [20]. Ownership of a mobile phone and access to the internet at least once a week was required for intervention delivery. Subjects had to be failing to meet one or more of the key behaviors for modification which were less than 2 serves fruit daily; less than 5 serves vegetables daily; more than 1 high-energy, high-fat take-out meal weekly; more than or equal to 1 liter SSBs weekly; less than 60 minutes of moderate physical activity daily. Exclusion criteria were pregnancy or planning to fall pregnant within 9 months, participation in an alternate weight loss program, weight loss of > 10 kg in preceding 3 months, medications that caused > 2 kg weight gain, disordered eating or medical contraindication, and non-English speaking [20].

The mHealth program ran for 12 weeks after which participants entered a maintenance intervention phase for an additional 6 months.

### Twelve-Week “TXT2BFiT” Program

The program promotes the consumption of core food groups and limitation of energy-dense nutrient-poor discretionary foods. Key behaviors for change are fruit and vegetable intake to meet recommended amounts (behavior 1); high-fat, high-energy take-out meals (behavior 2) and SSBs (behavior 3) are discouraged. In addition, participants are encouraged to achieve 60 minutes of physical activity daily (behavior 4), the upper level of Australian recommendations [23]. Participants allocated to the treatment arm received a multicomponent mHealth program. This included 5 coaching calls by a dietitian skilled in motivational interviewing. Goal setting and review were included in the coaching and modeled on control theory with their intake (input function) compared with recommended (comparator) and, therefore, provided feedback to improve their behavior (week 0, 2, 5, 8, 11) [24]. For each of the 4 key behaviors addressed, a staging algorithm based on the transtheoretical model was completed as part of the baseline survey by all participants [20,25]. This was used to generate a personalized set of messages (8 messages a week) from our bank of text messages to be sent over the 12-weeks. Messages were stratified by sex and whether the participant was in pre-contemplation, contemplation, preparation, action, or maintenance stages for each of the 4 behaviors. More cognitive messages were included if a behavior was in the early stage for change and messages were more behavioral if participant was in the action or maintenance stages for any given behavior. Twelve emails (once a week) were sent by the dietitian who offered coaching and repeated the information in the text messages with links to remind participants to use the other resources provided. After a coaching call, the goals set were reiterated in the emails sent by the dietitian.

Other components of the program were a comprehensive 18-page diet and nutrition booklet with physical activity guidelines and a website. This website gave access to 4 designer mobile phone apps for education and self-monitoring for each of the 4 key lifestyle behaviors addressed. Other resources were online weight tracker, printable charts such as “eating on a budget,” “emergency meal tool kit,” “meal planner,” “seasonal guide to fruit and vegetables,” “tips for take-outs,” physical activity planner and “staying healthy over holidays;” and a blog facility for communication [26].

### Control Program

A minimal intervention was delivered to controls, which included 4 text messages, 1 on each key behavior, over 12 weeks (fruit and vegetables, take-out meals, SSBs, and physical activity). Control participants also received a 2-page handout based on the Australian dietary guidelines and physical activity guidelines. They had access to a website (separate from the password-protected website of the intervention participants) that contained only the participant information sheet and the 2-page handout. An introductory phone call was made to each participant but no coaching was provided.

### Six-Month Maintenance Phase

After the 12-week “TXT2BFiT” program, intervention participants received a low dose maintenance intervention. This consisted of monthly text messages and emails, and participants had continued access to the website. Two booster coaching phone calls at 5 and 8 months from baseline were included. Control participants had no further treatment contact during this period.

### Measurements

The primary outcome was change in weight. All participants were weighed to the nearest 0.1 kg and had their height measured to the nearest 0.1 cm by their primary care physician at baseline according to a standard protocol. Participants were invited to be weighed by study personnel at the end of the 12-week trial [20]. Self-reported measures of body weight were collected at baseline, end of 12-week trial, and again at the end of 6-month maintenance (9 months) via the Web-based survey instrument. BMI was calculated. Following a standardized procedure, participants were provided with instructions on self-weighing by the dietitian.

Secondary outcomes were assessed using online surveys at baseline, the end of the 12-week trial, and the end of the 6-month maintenance. These included changes in fruit and vegetable intake (daily servings), SSBs (weekly intake), and weekly frequency of take-out meals assessed using short categorical questions. Change in frequency and minutes of physical activity were assessed using the short-form International Physical Activity Questionnaire (IPAQ). Details of the questions have been published previously [20]. A $AU10.00 gift voucher was given for completion of each survey and clinic attendance for weight measurement.

Demographic details were collected via the online questionnaire that included age, sex, postcode used to determine socioeconomic status (SES), language spoken at home, and the WHO-5 well-being questionnaire [20,27]. The delivery of the program was monitored by number of coaching calls completed, number of emails and text messages delivered, and number of downloads of the mobile phone apps. Participants in the intervention were asked to reply to 22 text messages over the 9 months (16 in the first 12 weeks and 6 in the next 6 months). Both the 12-week and 9-month online surveys included questions on use of the program elements.

### Sample Size Calculation

The sample size was calculated based on a difference of 2 kg between intervention and control groups, allowing for a standard deviation of 10 kg and a correlation of 0.8 of baseline weight and final weight. With 142 subjects in each arm, this difference could be detected with 80% power at *P*<.05 (two sided). To allow for a 20% drop out rate, the sample size was increased to 354 in total. As stated above, recruitment was ceased at 250 participants [21].

### Statistical Analysis

Attrition bias was examined using *t* tests for continuous variables and chi-square tests for categorical variables. The baseline characteristics of completers and non-completers at 9 months within both the intervention and control groups were compared. The IPAQ was scored using standard methods to yield a continuous measure of reported physical activity minutes weekly (metabolic equivalent of task, MET-min) [28]. Differences between the experimental and control group over time in the continuous variables, such as body weight, BMI, physical activity, and WHO-5 outcomes, were estimated using linear mixed models, with an unstructured correlation matrix, adjusted for sex and primary care practice (fitted as fixed effects) and implemented with PROC MIXED. We examined plots of panel-studentized residuals which demonstrated normality and constant variance. Interaction between time and group was included in the model. Diet outcomes (fruit, vegetables, SSBs, and take-out meals) were analyzed using cumulative logistic regression models with general estimating equations (GEE) to account for correlation between time points and multiple imputations to account for missing values. Ten imputed data sets were created using chained equations utilizing baseline values and available data at 3 and 9 months, as well as participant baseline characteristics including sex, ethnic background (language spoken at home), recruitment practice, and allocation. This included odds of improvement in diet-related behaviors and odds of meeting suggested intakes of 2 serves fruit, 3 or more serves of vegetables, less than 500 mL of SSBs per week, and less than one take-out meal weekly. The effect of missing data was investigated as part of a sensitivity analysis using multiple imputation under the missing not at random (MNAR) assumption by searching for a tipping point that reverses the primary outcome conclusion [29]. Clinically plausible weight gains (fixed values) were added to randomly generated imputed values to investigate the impact at 3 month time-point and 9 month end point. Ten imputed data sets were created as described above. All analyses were performed with SAS (version 9.2 SAS Institute Inc. Cary NC, USA).

## Results

**Figure 1:**
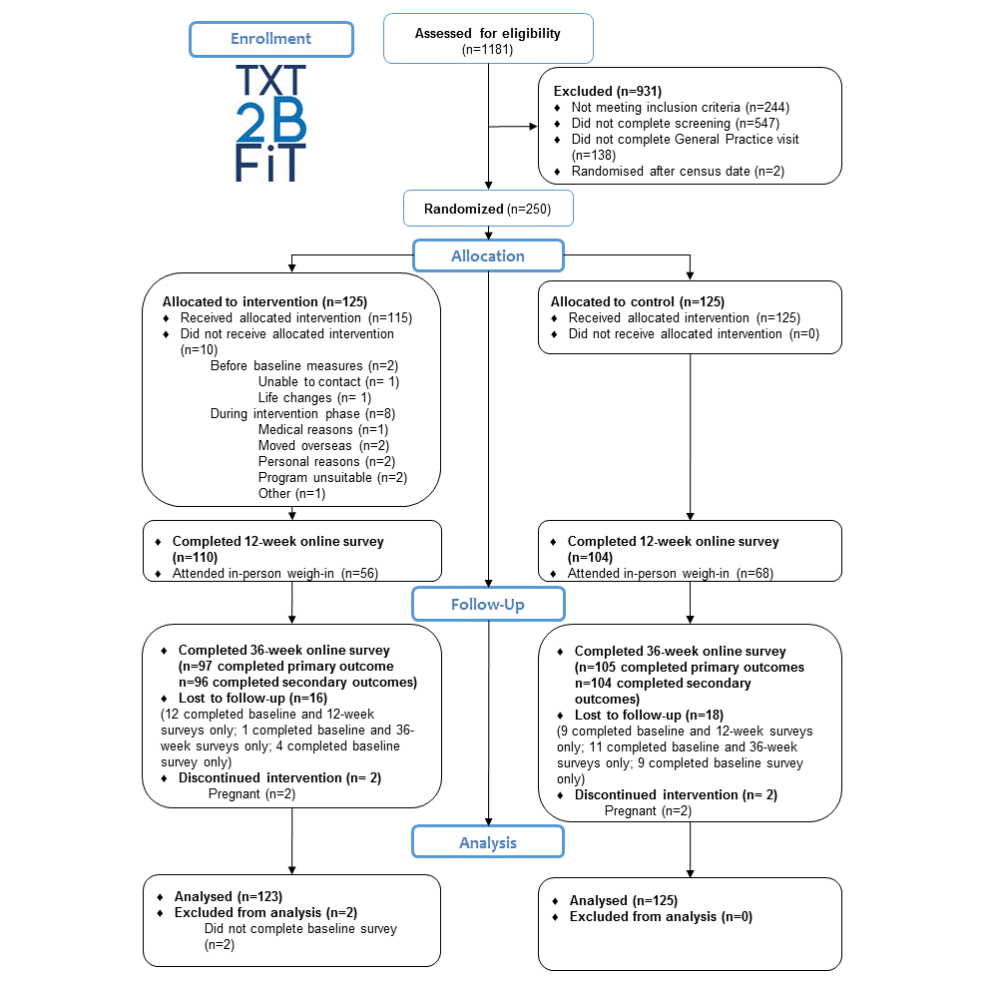
Flow of participants through the 9-month trial.

Figure 1 displays the flow of participants through the trial. In all, 1181 attempts of the screener survey were recorded with 547 of these failing to complete screening. An additional 244 failed to meet the inclusion criteria and 138 eligible participants failed to complete the visit to the primary care physician and were not randomized. Two participants were randomized after census date.

One hundred and twenty-five participants were allocated to each arm. After the 12-week program, 110 intervention participants and 104 control participants completed the Web-based survey for assessment of outcomes. At completion of the maintenance phase, 97 intervention participants and 105 control participants completed the final online survey.

Table 1 summarizes the demographic characteristics and lifestyle behaviors of the population at baseline. Participants were mostly in the overweight BMI range, of higher SES, from English-speaking backgrounds, and tertiary educated. Approximately 3 in 5 participants were females and mean WHO-5 was just below middle of the scale of 25, indicating a tendency of poor well-being. Most participants failed to meet the criteria for fruits (2 serves), vegetables (3 serves), and take-out meals (more than 1 per week). Most participants consumed less than 500 mL of SSBs weekly and the mean total daily physical activity was approximately 55 minutes of moderate physical activity on each of the past 7 days; 51.6% met the national recommendation for 30 minutes of physical activity per day (48.8% intervention, 54.4% control). A comparison of those participants retained in the study versus those lost to follow-up after 9 months showed no significant differences by demographic characteristics neither for intervention nor for the control group.

**Table 1:** Characteristics and baseline behaviors of participants in the TXT2BFiT trial.

Characteristic		Intervention group (n=123)^a^ mean (SD) or n (%)	Control group (n=125) mean (SD) or n (%)
**Age in years, mean (SD)**		28.1 (4.9)	27.2 (4.9)
**Gender, n (%)**			
	Female	73 (59.3)	79 (63.2)
**Weight status**			
	Normal BMI 23.0-24.9	24 (19.5)	33 (26.4)
	Overweight BMI 25.0-29.9	83 (67.5)	70 (56.0)
	Obese BMI 30.0-31.99	16 (13.0)	22 (17.6)
**BMI (kg m^-2^), median (IQR)**		27.1 (3.7)	26.8 (4.2)
**WHO-5 score**		11.8 (4.7)	12.9 (4.5)
**SES^b^, n (%)**			
	0-60	8 (6.5)	7 (5.6)
	61-80	28 (22.8)	17 (13.6)
	81-100 (highest)	87 (70.7)	101 (80.8)
**Ethnic background, n (%)**			
	English speaking	82 (66.7)	90 (72.0)
	Other^c^	41 (33.3)	35 (28.0)
**Education, n (%)**			
	High school or below	27 (22.0)	21 (16.8)
	Some tertiary education	22 (17.8)	25 (20.0)
	University degree	74 (60.2)	79 (63.2)
**Fruit**	< 2 serves per day	82 (66.7)	77 (61.6)
**Vegetable**	≤ 3 serves per day^d^	104 (84.6)	107 (85.6)
**SSB** ^e^	≥ 500 mL per week	37 (30.1)	44 (35.2)	
**Take-out meals**	> once per week	75 (60.9)	79 (63.2)
**Physical activity**	Total MET^f^-mins weekly	1620 (1581)	1647 (1475)

^a^Two participants had measured variables but did not complete baseline self-report survey.

^b^SES: socioeconomic status by quintile with the bottom three quintiles collapsed into one.

^c^European, Asian, Pacific Islander, and Arabic ethnicities collapsed.

^d^Australian recommendations are 5 serves per day but the World Health Organization recommendation of 3 is used here.

^e^SSB: sugar-sweetened beverages.

^f^MET: metabolic equivalent of task.

Table 2 presents body weight, BMI, and MET-minutes of physical activity per week for intervention and control groups. After the 12-week program the intervention participants weighed 3.7 kg (95% CI −6.1 to −1.3, *P*=.003) less than controls and after maintenance, 9 months from baseline, the intervention group weighed 4.3 kg (95% CI −6.9 to −1.8) less than controls (*P*=.001). The changes in BMI equated to a difference of 0.56 kg/m^2^ (95% CI −1.22 to 0.09, *P*=.093) after the 12-week program and 0.78 kg/m^2^ (95% CI −1.53 to −0.02, *P*=.044) at end of maintenance stage. Sensitivity analyses outlined above generated consistent findings for the primary outcome, with the addition of up to 2.4 kg at 3 months and 7.2 kg at 9 months to the imputed values. Although the intervention improved their moderate physical activity by 12 minutes per day more than the controls at 12 weeks, the differences were not significant and disappeared by 9 months. The WHO-5 score showed improvement in both groups with no significant differences between them. The mean increase in both groups was clinically meaningful, with the mean score above the cut point of 13 indicating improved well-being.

Odds ratios comparing the odds for improving intake of fruit, vegetables, SSBs, and take-out meals for intervention and control groups are reported in Table 3. After the 12-week program, the odds that the intervention group improved intake compared to the control group were significantly greater for vegetables (*P*=.006), SSBs (*P*=.024), and take-out foods (*P*=.013). At the conclusion of maintenance stage, the intervention group had greater odds of maintaining improvements in all 4 diet variables. At the end of the program and the end of maintenance, the intervention group had greater odds of meeting suggested intakes for all dietary variables (Table 4).

**Table 2:** Comparison of self-reported weight, BMI^a^, and physical activity in intervention (n=123) versus control (n=125) at baseline, end of program (3 months), and end of maintenance periods (9 months).

	Baseline=0 months, mean (SD)^b,c^		End of program=3 months, mean difference (SD)^c^			End of maintenance=9 months, mean difference (SD)^c^		
	TXT2BFiT	Control	TXT2BFiT	Control	Model β (95% CI)	TXT2BFiT	Control	Model β (95% CI)
Weight, kg					
	78.4 (11.2)	79.3 (12.6)	−2.2 (3.1)	−0.23 (2.3)	−3.7 (−6.1 to −1.3) *P*=.003	−3.8 (4.9)	−0.80 (3.7)	−4.3 (−6.9 to −1.8) *P*=.001
BMI, kg/m^2^					
	27.3 (2.3)	27.0 (2.7)	−0.76 (1.0)	−0.08 (0.78)	−0.56 (−1.22 to 0.09) *P*=.093	−1.30 (1.7)	−0.26 (1.28)	−0.78 (−1.53 to −0.02) *P*=.044	
Physical activity, MET-min					
	1620 (1581)	1647 (1475)	625 (1932)	302 (1411)	333 (−206 to 871) *P*=.225	872 (1918)	797 (2115)	70 (−474 to 614) *P*=.801
Physical activity, days					
	6.6 (3.3)	7.4 (3.8)	2.1 (3.8)	0.5 (3.7)	1.0 (0.0 to 2.0) *P=*.050	2.1 (4.3)	1.3 (4.4)	0.2 (−0.8 to 1.3) *P=*.679	
WHO-5 score					
	11.8 (4.7)	12.9 (4.5)	3.2 (4.7)	1.2 (4.7)	0.9 (−0.4 to 2.1) *P*=.176	3.4 (4.5)	1.4 (5.2)	0.8 (−0.5 to 2.1) *P*=.202

^a^BMI: body mass index.

^b^SD: standard deviation.

^c^Mean difference between groups (95% confidence intervals) adjusted for practice and sex

Monitoring of intervention delivery showed that 92% of coaching calls were completed during the 12-week program and 81% during 6-month maintenance period. All emails were delivered and for the text messages, 98% were delivered during the first 12 weeks and 96% during the 6-month maintenance. During the 12-week program, 53% of participants replied to more than half the text messages but only 40% replied during the maintenance phase. All participants reported using the text messages at 12 weeks and 60% at 9 months. Fifty-two percent of participants downloaded the physical activity app, 43% downloaded the fruit and vegetable app, and 31% the beverages app [30]. The data on downloads of the take-out app could not be determined. The company hosting the website made changes that lead to a temporary loss of data from this app but it did not affect the other three.

**Table 3:** Odds ratios (95% confidence intervals) of improved intakes^a^ for TXT2BFiT intervention versus control post-intervention (3 months) and post-maintenance (9 months) adjusted for practice and sex.

Phase		Fruit^a^	Vegetables^a^	Sugar-sweetened beverages^a^	Take-out meals^a^
**Post intervention, time=3 months**					
	Control	1.00 (reference)	1.00 (reference)	1.00 (reference)	1.00 (reference)
	TXT2BFiT	1.31 (0.79-2.15) *P*=.292	2.03 (1.23-3.35) *P*=.006	1.67 (1.07-2.61) *P*=.024	2.16 (1.18-3.95) *P*=.013
**Post maintenance, time=9 months**					
	Control	1.00 (reference)	1.00 (reference)	1.00 (reference)	1.00 (reference)
	TXT2BFiT	2.38 (1.41-4.01) *P*=.001	1.94 (1.19-3.16) *P*=.008	1.74 (1.10-2.77) *P*=.018	1.98 (1.17-3.34) *P*=.010

^a^Odds ratios were estimated using proportional odds models. Lower odds ratios, but greater than 1, were observed for the most improved categories.

**Table 4:** Odds ratios (95% confidence intervals) of meeting recommendations for TXT2BFiT intervention versus control post-intervention (3 months) and post-maintenance (9 months) adjusted for practice and sex.

Phase		Fruit ≥ 2 serves	Vegetables ≥ 3 serves	Sugar-sweetened beverages < 500 mL per week	Take-out meals < one per week
**Post intervention time=3 months**					
	Control	1.00 (reference)	1.00 (reference)	1.00 (reference)	1.00 (reference)
	TXT2BFiT	1.84 (1.01-3.34) *P*=.046	2.05 (1.16-3.62) *P*=.014	4.77 (1.96-11.62) *P*=.001	2.37 (1.21-4.63) *P*=.012
**Post 6 maintenance time=9 months**					
	Control	1.00 (reference)	1.00 (reference)	1.00 (reference)	1.00 (reference)
	TXT2BFiT	3.83 (2.10-6.99) *P*=.001	2.42 (1.32-4.44) *P*=.005	3.11 (1.47-6.59) *P*=.003	1.88 (1.07-3.30) *P*=.028

## Discussion

This is one of the first trials of a multicomponent mHealth program for delivery to young adults with demonstrated maintenance of weight management and nutrition-related behavior change after the 12-week program. As hypothesized, the TXT2BFiT program prevented weight gain [25] leading to weight loss that can be maintained after 6 months follow-up with minimal support. The use of mHealth to deliver effective lifestyle health promotion shows promise to halt the rising incidence of obesity during young adulthood—a group recognized as difficult to reach. The ubiquitous use of mobile phones by this age group allows for a number of communication channels to be used to deliver multicomponent programs.

The 12-week TXT2BFiT program led to additional positive health benefits through maintenance of improved diet behaviors at 9 months from baseline. This is the likely explanation for the 6-month maintenance of weight loss. In addition, increasing fruit and vegetable intake has benefits beyond weight management, in protection against cardiovascular disease and stroke and certain cancers [31-33]. In the past decade, consumption of SSBs has been associated with not only weight gain but also cardiovascular disease and type 2 diabetes [34]. The age group targeted here is the largest consumer of fast food meals among adults [35]. Such food is inevitably high in deleterious nutrients such as saturated fat and sodium, and higher frequency of intake is associated with type 2 diabetes in Australian young adults [36]. The intervention failed to produce increases in physical activity compared with control. Both groups appeared to have higher amounts of physical activity at 3 and 9 months but with wide variation. In previous research in a similar group of younger adults, 75% reported at least 150 minutes of moderate physical activity per week but most wanted to increase their physical activity [37]. This is higher than that in the current trial, with 51.6% of participants meeting national physical activity recommendations. The controls were sufficiently motivated to enroll in the study and likely had the capability and opportunity to increase their physical activity without intervention input. It is also of note that the controls did not gain weight during the 12-week program or at 9 months.

A number of other trials using new technologies have been conducted in young adults in the United States [17]. The CITY study compared treatment using 6 face-to-face group sessions followed by monthly phone calls for 24 months (PC), with both an intervention delivered via a mobile phone app and a control group [38]. Weight measurements in the 18- to 35-year-olds indicated a significant loss by the PC group compared with control and app group at 6 months but no difference between app and control groups. By 12 months, all differences disappeared [38].

This study was different from other studies in that the current intervention included 5 short coaching calls in the 3-month intensive phase as a component of the mHealth intervention. In our former pilot RCT to assess the feasibility of delivering the intervention in 50 young adults, we found no difference in weight loss between groups at 12 weeks because both reduced their weight [39]. The extra communication component in this study was the addition of short coaching calls. This allowed more personalized feedback for goal setting and review whereby their performance against a recommended behavior such as 2 daily serves of fruit was used to set goals to work toward the achievement of the target intake. It adds an additional cost to the program that amounted to approximately AU $45 per participant and cost-effectiveness comparisons with totally electronically delivered and traditional face-to-face intervention warrant further research. However, as discussed below, few electronic or entirely app-based interventions demonstrate effectiveness.

Other studies in young adults have used Web-based or email programs. Kattelmann et al delivered a 10-week Web-based intervention to 1639 US college students addressing healthy eating, physical activity, stress, and weight management in an RCT, and assessed post-intervention effects and 12-month maintenance. While both diet and physical activity behaviors improved, no changes in weight occurred and the effects were not maintained 12 months later [40]. Schweitzer et al delivered an adaptation of A Lifestyle Intervention via Email, the ALIVE program, for 24 weeks in a pilot RCT to 148 college students aged 18-20 years. While no differences in body weight were found, the intervention participants increased their intake of fruits as a snack and marginally decreased the percentage energy from saturated fat [41]. Park et al conducted a Web-based RCT in 160 US students aged 18-24 years comparing tailored advice based on the transtheoretical model of behavior change with non-tailored advice and found no differences in fruit and vegetable consumption [42]. This could suggest that using multiple components in mHealth such as text messaging and coaching calls provide a more personalized approach than Web-based techniques that young adults find helpful for changing their behaviors. The college-based RCT by Gow et al with four treatments including no intervention, 6 weeks of a Web-based intervention, 6 weeks of feedback on weight and calorie intake, and a combination of Web-based intervention with weight and calorie feedback found that the combined group attained the lowest BMI [43]. This further illustrates the advantages of feedback communication in addition to Web resources. Bertz et al studied 167 first-year US college students providing Wi-Fi scales and emailed graphs of weight to show changes compared with no feedback control group. It was found that regular weighing with feedback prevented weight gain [44]. Thus, the importance of building education and counseling with feedback along with ongoing monitoring and self-monitoring into a mHealth program is apparent. The inclusion of a range of demonstrated theory-based behavior change techniques, as in this study, also should be central to any mHealth program [45].

The most successful Web-based intervention to date was conducted in Scotland. Nikolaou et al randomized 20,975 university students to a 40-week three-arm RCT: control and two treatments [46]. While the control group gained weight (mean 2.0 kg, 95% CI 1.5-2.3), both treatment groups lost weight: treatment one −1.0 kg (95% CI −1.3 to −0.5) and treatment two −1.35 kg (95% CI −1.4 to −0.7). Both interventions were novel in their approach. The first treatment was based on the “rational” model that individuals when presented with information will make the best use of it. The messages overtly addressed the problem of weight gain and obesity. The second intervention was by “stealth” and raised discussion around social and political movements associated with food and health and obesity. For 19 weeks, participants would log into the Web-based modules to be completed weekly. The advantage of this study is that it was embedded within the university learning environment and was advertised as a new course being tested. Many undergraduate students participated after invitation, unlike the other studies that either recruited volunteers from within the college environment or the population at large. While this intervention could likely be adapted for other college students, whether it would be successful when participants must be recruited from the general community of young adults is uncertain.

One of the strengths of this study is that the age range of recruited participants was well distributed between 18 and 35 years. The program reached a greater proportion of males (40%) than usual in weight management studies [10] and included 30% participants who were not born to an English speaking family. However, our results may not be generalizable to all young adults because the study group tended to be of a higher SES and messaging might be country specific.

### Limitations

A perceived limitation is that the study maintenance phase was for 6 months only, that is 9 months from baseline, whereas up to 2 years is suggested for weight maintenance [47]. However, maintenance of nutrition behaviors with habit formation may be developed in shorter time frames [48]. Another limitation is the assessment of outcomes using self-report. While there was no difference in the comparison of weight change between both groups when measured and self-reported weight was used at 12 weeks [26], we cannot be certain if this remained the case at 9 months from baseline. The IPAQ may not be sufficiently sensitive to monitor changes in physical activity [28]. Although acknowledged as a limitation, the use of an objective measure like biomarkers is not practical for a remotely delivered intervention and costs of providing Wi-Fi enabled scales, from which data can be accessed, may prove too costly for such an intervention if it is to be scaled up in the population. Recruiting young adults to participate in lifestyle intervention proved challenging in this study. Both the primary care physicians and their patients had a lower than expected uptake of the program [21]. Costs, time, and methods of recruitment require consideration when planning replication trials or scale-up and roll out. Further consideration should be given to recruiting a larger sample size to determine the effects on well-being because, while no evidence was provided in this study, the 95% confidence intervals include beneficial values. Finally, the study had multiple components and we did not attribute the changes to any component in isolation. While the 12-week findings from our study were included in a recent meta-analysis of mobile phone apps for weight loss, it can be seen that less than half the sample downloaded the apps [49].

### Conclusion

In conclusion, delivery of an mHealth theory-based intervention for healthy lifestyle and prevention of weight gain in 18- to 35-year-olds was effective in achieving and maintaining weight loss and improved diet behaviors. Countries such as the United States, United Kingdom, and Australia are recognizing that programs with wide reach but of low cost are required in young adulthood [18]. Although the messaging component of the mHealth program was developed in the local context, the behaviors targeted are globally problematic with SSB consumption, high-fat, high-energy take-out meals, and poor vegetable intakes prominent in young adults in the United States [18]. Given the potential for universal adoption and wide reach in this age group, we suggest replication trials of mHealth be conducted in a broader range of young adults throughout countries battling obesity.
